# Limited agreement between nutritional screening tools and CT-defined body composition alterations in hospitalized cancer patients: a real-world analysis

**DOI:** 10.1007/s00520-026-11086-y

**Published:** 2026-08-08

**Authors:** Matheus de Souza Lima, Almir Galvão Vieira Bitencourt, Thais Manfrinato Miola

**Affiliations:** AC Camargo Cancer Center, São Paulo, Brazil

**Keywords:** Cancer, Malnutrition, Sarcopenia, Body composition, Computed tomography, Nutritional screening

## Abstract

**Purpose:**

Nutritional screening tools are widely used in routine oncologic care to identify patients at nutritional risk. However, their ability to identify hospitalized patients with relevant alterations in body composition remains unclear. This study aimed to evaluate whether nutritional screening tools routinely used in clinical practice capture patients with CT-defined changes in muscle mass and quality.

**Methods:**

This retrospective, cross-sectional study included hospitalized adult cancer patients treated at a tertiary cancer center in São Paulo, Brazil, between 2022 and 2023. Eligible patients underwent abdominal computed tomography (CT) and nutritional screening during hospitalization. Skeletal muscle index (SMI) and skeletal muscle density (SMD) were assessed at the third lumbar vertebra (L3) using CoreSlicer® software. Nutritional risk was evaluated using routinely implemented screening tools, and associations with CT-derived body composition parameters were analyzed.

**Results:**

Two hundred seventy-two patients were included. Low muscle mass was identified in 51.1% of patients and low muscle density in 52.2%, including individuals with normal or elevated body mass index. Nutritional risk identified by NRS-2002 was significantly associated with SMI but showed limited ability to identify CT-defined alterations when used in isolation. The CNS demonstrated high sensitivity but low specificity. Many patients with CT-detected alterations in muscle mass and quality were not classified as at nutritional risk by screening tools.

**Conclusion:**

Widely used screening instruments may overlook clinically relevant alterations in body composition among patients. When imaging is already available as part of routine oncologic care, opportunistic CT-based assessment may complement screening and support more individualized supportive care interventions.

## Introduction

Malnutrition and loss of skeletal muscle mass are highly prevalent among cancer patients and are consistently associated with poor prognosis, increased morbidity, greater treatment-related toxicity, and reduced quality of life. These alterations result from a complex interaction between tumor burden, systemic inflammation, and anticancer therapies. Up to 80% of patients experience weight loss or malnutrition during the disease course, and approximately 10–20% of cancer-related deaths are attributed to nutritional deterioration rather than tumor progression itself. Despite its clinical relevance, cancer-related malnutrition remains underdiagnosed and undertreated worldwide [1–6].

International guidelines recommend routine nutritional screening for all cancer patients as a strategy to identify individuals at risk and guide further assessment. The European Society for Clinical Nutrition and Metabolism (ESPEN) emphasizes that effective screening tools should be rapid, low-cost, sensitive, and specific, recommending validated instruments such as the Nutritional Risk Screening-2002 (NRS-2002). However, low muscle mass and impaired muscle quality are strongly associated with adverse outcomes and may remain undetected, particularly in patients with preserved or elevated body mass index. Computed tomography (CT) has emerged as a reference method for body composition assessment in oncology, with cross-sectional imaging at the third lumbar vertebra (L3) demonstrating strong correlation with whole-body muscle and adipose tissue [7–12].

Previous studies comparing nutritional screening tools with CT-derived muscle mass have demonstrated substantial discordance. In a cohort of 725 chemotherapy outpatients, up to 61% of patients with sarcopenia identified by CT were classified as having low nutritional risk by screening instruments. These findings underscore that while screening tools are essential to guide further evaluation, they were not designed to detect alterations in body composition, and no consensus exists regarding the optimal screening strategy in oncology [13].

In Brazil, nutritional screening tools are frequently the first and, in many institutions, the only step used to identify nutritional risk in hospitalized cancer patients. In routine clinical practice, patients are typically referred for comprehensive nutritional assessment only when they are classified as at nutritional risk by these screening instruments, whereas those screened as “no risk” often do not undergo further evaluation. This workflow may result in under recognition of clinically relevant nutritional impairment.

This limitation suggests that nutritional screening instruments used in routine clinical practice may fail to identify a relevant proportion of patients with underlying muscle depletion or impaired muscle quality.

Therefore, this study aimed to evaluate the association between commonly used nutritional screening tools and CT-derived measures of muscle mass and muscle quality in hospitalized cancer patients.

## Materials and methods

Clinical and clinicopathological data were obtained from the A.C. Camargo Cancer Center database for patients hospitalized between January 2022 and December 2023. Eligible participants were adults aged ≥18 years with a confirmed cancer diagnosis who had undergone abdominal CT within 30 days prior to admission, reflecting routine imaging intervals in oncologic care, and had documented nutritional screening during hospitalization. Patients admitted to the intensive care unit or receiving exclusive palliative care were excluded. In cases of multiple hospitalizations, only the first admission was considered.

**Fig. 1:**
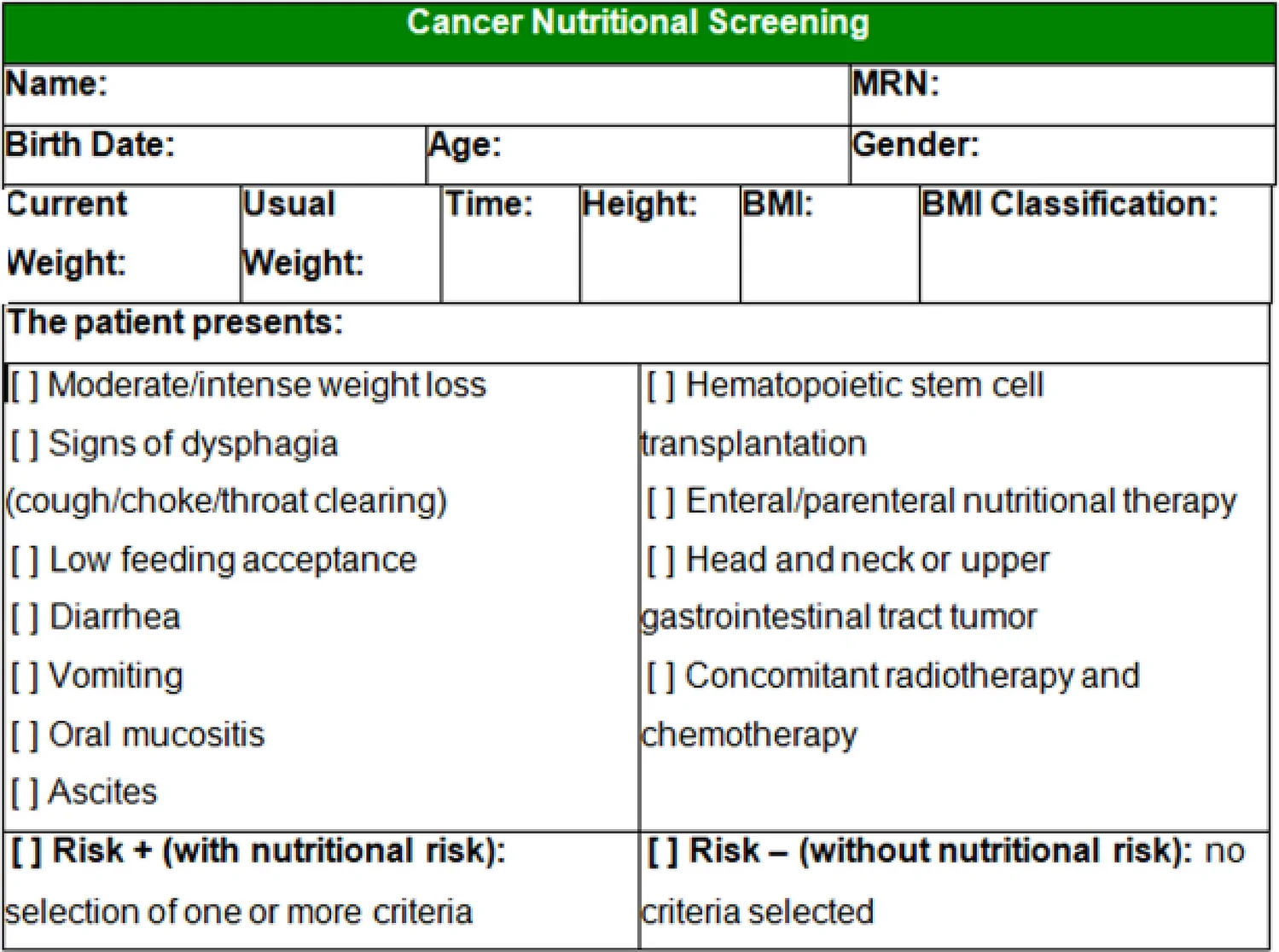
Cancer nutritional screening

Nutritional risk was assessed using the Nutritional Risk Screening-2002 (NRS-2002) and the Nutritional Screening Tool (CNS), reflecting routine institutional practice. The CNS (Fig. 1) is an institutional dichotomous screening instrument that considers variables related to food intake, percentage of weight loss, cancer diagnosis, treatment characteristics, and nutrition-impact symptoms. The screening includes the following criteria: moderate/intense weight loss, signs of dysphagia, low feeding acceptance, diarrhea, vomiting, oral mucositis, ascites, hematopoietic stem cell transplantation, enteral/parenteral nutritional therapy, head and neck or upper gastrointestinal tract tumors, and concomitant radiotherapy and chemotherapy. Patients were classified as “Risk” when at least one criterion was present and as “No Risk” when no criteria were identified. The CNS does not generate a cumulative numerical score and was applied as a rapid screening tool for identifying patients requiring nutritional attention.

Body mass index (BMI) was calculated according to World Health Organization criteria for adults and Pan American Health Organization criteria for older adults [14].

Body composition was evaluated using a single axial CT slice at the inferior border of the third lumbar vertebra (L3), analyzed with CoreSlicer® software. Skeletal muscle area, including psoas, paravertebral, and abdominal wall muscles, was quantified using semi-automated segmentation and manually corrected when necessary. Skeletal muscle was identified within a range of −29 to + 150 Hounsfield Units. Skeletal muscle index (SMI, cm^2^/m^2^) was calculated by normalizing muscle area to height. Muscle depletion was defined as SMI < 55 cm^2^/m^2^ for men and <39 cm^2^/m^2^ for women. Mean skeletal muscle density (SMD) was measured using established sex-specific cut-offs [7, 15]. All CT analyses were performed by trained investigators following standardized protocols.

### Statistical analysis

Statistical analyses were performed using IBM SPSS Statistics, version 26.0 (IBM Corp., Armonk, NY, USA). Continuous variables were compared using Student’s *t*-test or Mann–Whitney *U* test, as appropriate. Categorical variables were analyzed using Pearson’s chi-square test or Fisher’s exact test, as appropriate. Multivariable logistic regression analyses were performed to assess independent associations between nutritional screening tools and CT-derived low muscle mass and low muscle quality. The models included NRS-2002 classification and CNS risk as variables of interest, with adjustment for age, BMI category, and presence of metastasis. Odds ratios (ORs) and 95% confidence intervals (CIs) were calculated. Statistical significance was set at *p* < 0.05.

## Results

**Fig. 2:**
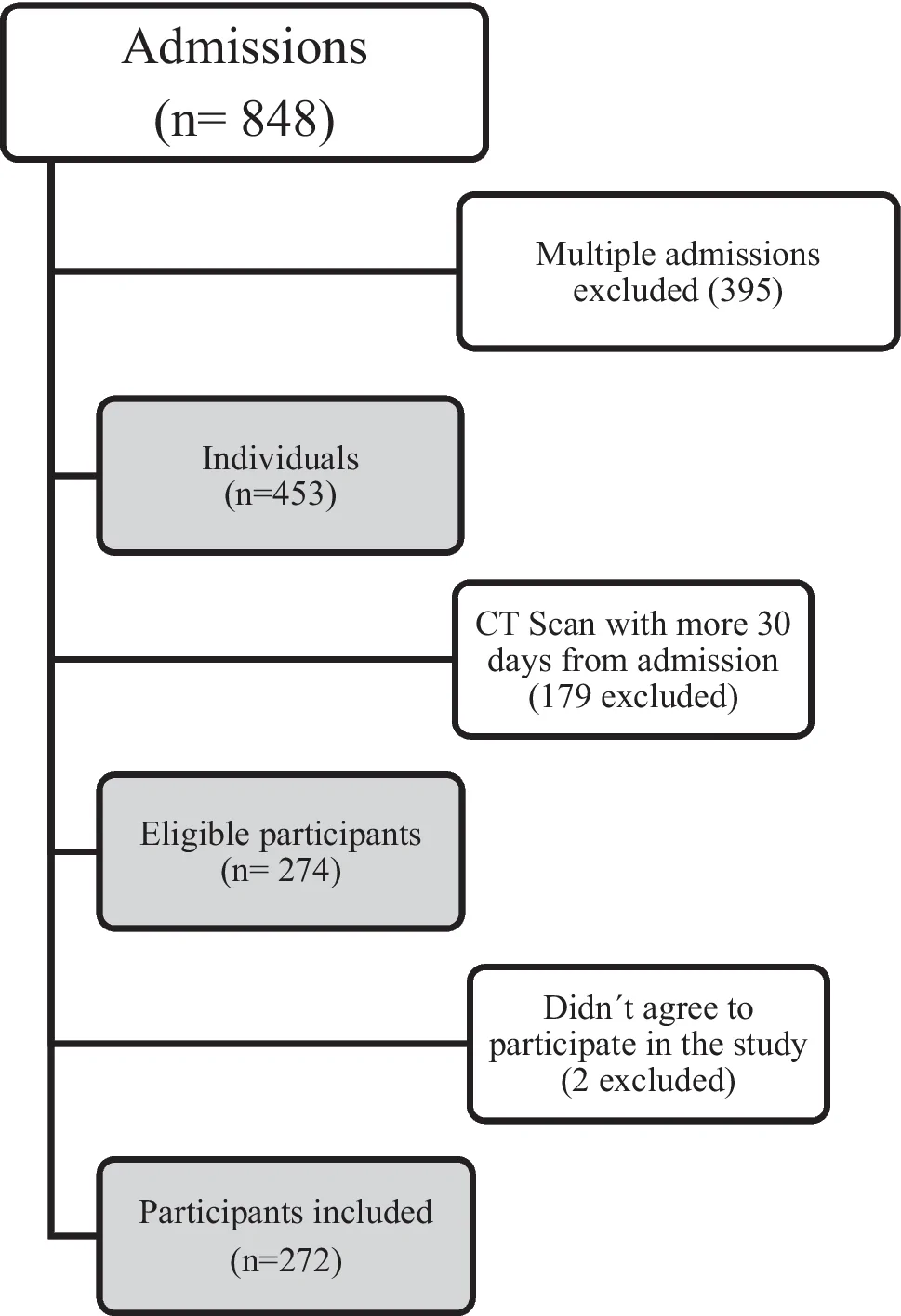
Participant eligibility flowchart

During the data collection period, 848 hospital admissions were recorded. For patients with multiple admissions within this timeframe, only the first hospitalization was considered, resulting in 453 unique individuals. After excluding patients who did not undergo computed tomography (CT) during the study period, 274 eligible participants remained. Of these, two declined to participate, yielding a final sample of 272 included participants (Fig. 2). The cohort was predominantly female (59.6%), with a median age of 62 years. The most common tumor sites were upper gastrointestinal (18.4%) and colorectal (12.5%). Most patients presented with non-metastatic disease (71.3%). Length of hospitalization ranged from 1 to 93 days (median: 6), and the majority of admissions were clinical (78.3%). Clinical and demographic characteristics are detailed in Table 1.

**Table 1:** Clinical and demographic characteristics

Variable	Category	*N* (%)
Gender	Male	110 (40.4)
	Female	162 (59.6)
Age (years)	Min–Max	20–93
	Mean/median	60.49/62
Tumor location	Upper gastrointestinal	50 (18.4)
	Colorectal	34 (12.5)
	Breast	34 (12.5)
	Hematological	29 (10.7)
	Urological	28 (10.3)
	Gynecological	23 (8.5)
	Lung and chest	19 (7)
	Head and neck	16 (5.9)
	Sarcoma and bone tumors	10 (3,7)
	Cutaneous	8 (2.9)
	Central nervous system	2 (0.7)
	Other	19 (7)
Metastasis	Yes	78 (28.7)
	No	194 (71.3)
Reason for hospitalization	Clinical	213 (78.3)
	Surgical	59 (21.7)
Length of hospital stay	Min–max	1–93
	Mean/median	9.58/6

Body weight showed wide variation, with median of 68 kg. Accordingly, BMI ranged broadly, with a median of 24.9 kg/m^2^. Skeletal muscle index (SMI) and skeletal muscle density (SMD) also varied considerably, with medians of 44.48 cm^2^/m^2^ and 33.38 HU (Table 2).

**Table 2:** Values of anthropometric parameters and muscle mass analysis by CT

Variable	Min–max	*N* (%)
Weight (kg)	25–129	-
BMI (kg/m^2^)	12,2–41,40	-
BMI (classification)	Underweight/malnutrition	58 (21.3)
	Normal range	111 (40.8)
	Overweight	53 (19.5)
	Obesity	50 (18.4)
CT muscle area (cm^2^)	69.11–245.44	-
SMI (cm^2^/m^2^)	26.01–81.45	-
SMI (classification)	Low	139 (51.1)
	Normal	133 (48.9)
SMD (HU)	1.5–56.35	33.11/33.38
SMD (classification)	Normal	130 (47.8)
	Low	142 (52.2)

Based on BMI, most patients were classified as well nourished (40.8%). However, SMI assessment revealed muscle mass depletion in 51.1% of participants, and SMD analysis showed low skeletal density in 52.2%.

Based on the combined NRS 2002 criteria, 55.1% of patients were classified as not at nutritional risk. According to CNS screening tool criteria, 73.2% of patients were classified as at nutritional risk. A significant association between SMI and nutritional risk was observed only with NRS 2002 (Table 3).

**Table 3:** Association between muscle mass index and nutritional screening tools

Variable	Category	SMI (*N*%)		*p* value
		Low	Normal	
NRS 2002	Risk	73 (52.5%)	49 (36.8%)	0.009
	No risk	66 (47.5%)	84 (63.2%)	
CNS	Risk	106 (76.3%)	93 (69.9%)	0.239
	No risk	33 (23.7%)	40 (30.1%)	

Associations between categorical variables were assessed using Pearson’s chi-square test

CNS screening demonstrated a sensitivity of 76%, specificity of 30%, positive predictive value (PPV) of 53%, negative predictive value (NPV) of 54%, and overall accuracy of 53% in relation to CT-defined low muscle mass. These findings suggest that while CNS is relatively effective in identifying patients at nutritional risk, its low specificity limits its ability to accurately rule out those not at risk, reducing its clinical utility when used in isolation.

For NRS-2002, sensitivity was 52%, specificity 63%, PPV 59%, NPV 56%, and overall accuracy 57% when compared with CT-defined muscle depletion. Although moderately specific in identifying patients not at risk, its limited sensitivity and overall performance indicate that NRS-2002 should be applied with caution and preferably in combination with additional clinical or laboratory assessments.

No significant association was found between skeletal muscle index (SMI) and skeletal muscle density (SMD) (*p* = 0.728). However, both NRS-2002 and CNS screening tools showed significant associations with SMD (*p* < 0.05) (Table 4).

**Table 4:** Association between skeletal muscle radiodensity (SMD), skeletal muscle index (SMI), and nutritional screening tools

Variable	Category	SMD (*N*%)		*p *value
		Low	Normal	
SMI	Low	74 (53.2)	65 (46.8)	0.728
	Normal	68 (51.1)	65 (48.9)	
NRS-2002	Risk	80 (56.3)	42 (32.3)	<0.001
	No risk	62 (43.7)	88 (67.7)	
CNS	Risk	116 (81.7)	83 (63.8)	0.001
	No risk	26 (18.3)	47 (36.2)	

Associations between categorical variables were assessed using Pearson’s chi-square test

Length of hospital stay did not differ significantly according to BMI (*p* 0.253), SMI (*p* 0.662), or SMD (0.127). In contrast, nutritional risk identified by both NRS-2002 and CNS screenings was significantly associated with longer hospitalization (*p* < 0.001). Patients classified as at risk by NRS-2002 had a mean stay of 13.02 days, compared to 6.69 days for those without risk. Similarly, CNS at-risk patients stayed an average of 11.11 days versus 5.44 days for non-risk patients.

Table 5 presents a multivariate logistic regression analysis using low muscle mass as the outcome; the NRS-2002 classification and CNS screening score were included as variables of interest, with adjustment for BMI (using the underweight BMI group as the reference category), age, and presence of metastasis. After adjustment, neither screening tool showed an independent significant association with the outcome: NRS-2002 (OR = 1.469; 95% CI 0.789–2.736; *p* = 0.225) and calculated risk (OR = 0.842; 95% CI 0.429–1.650; *p* = 0.616). Likewise, age (OR = 0.999; 95% CI 0.981–1.017; *p* = 0.898) and metastasis (OR = 0.640; 95% CI 0.357–1.146; *p* = 0.133) were not associated with the outcome. BMI, included as an adjustment variable, showed a significant association with the outcome in the model (*p* < 0.001). Compared with the reference group, patients with progressively higher BMI categories had lower odds of low muscle mass: normal weight (OR = 0.358; 95% CI 0.167–0.769; *p* = 0.008), overweight (OR = 0.156; 95% CI 0.064–0.376; *p* < 0.001), and obesity (OR = 0.129; 95% CI 0.051–0.327; *p* < 0.001).

**Table 5:** Low SMI multivariate logistic regression analysis

Variable	**OR**	**95% **CI	***p***
NRS-2002 risk	1.469	0.789–2.736	0.225
CNS risk	0.842	0.429–1.650	0.616
Age (years)	0.999	0.981–1.017	0.898
Metastasis	0.640	0.357–1.146	0.133
BMI			<0.001
Underweight/malnutrition	1.000	—	—
Normal range	0.358	0.167–0.769	0.008
Overweight	0.156	0.064–0.376	<0.001
Obesity	0.129	0.051–0.327	<0.001

*OR* odds ratio, *95% CI* confidence interval

*p*-values were derived from Wald tests

In the multivariate logistic regression analysis using low muscle quality as the outcome (Table 6), the same variables of interest were included in the model. Neither screening tool demonstrated an independent significant association with the outcome: NRS-2002 (OR = 1.743; 95% CI 0.902–3.369; *p* = 0.098) and CNS (OR = 1.494; 95% CI 0.729–3.063; *p* = 0.273). Metastasis was also not associated with the outcome (OR = 0.926; 95% CI 0.496–1.729; *p* = 0.810). Age showed an independent significant association with low muscle quality (OR = 1.081; 95% CI 1.055–1.108; *p* < 0.001), indicating that each additional year of age increased the odds of low muscle quality by approximately 8%. Regarding BMI, although the overall test for the variable did not reach statistical significance (*p* = 0.056), two BMI categories demonstrated an independent significant association with low muscle quality compared with the reference group (underweight): overweight (OR = 3.156; 95% CI 1.251–7.962; *p* = 0.015) and obesity (OR = 3.146; 95% CI 1.212–8.166; *p* = 0.018). The normal weight group showed a non-significant trend (OR = 1.926; 95% CI 0.877–4.231; *p* = 0.102).

**Table 6:** Low muscle quality multivariate logistic regression analysis

Variable	**OR**	**95% **CI	***p***
NRS-2002 risk	1.743	0.902–3.369	0.098
CNS risk	1.494	0.729–3.063	0.273
Age (years)	1.081	1.055–1.108	<0.001
Metastasis	0.926	0.496–1.729	0.810
BMI			0.056
Underweight/malnutrition	1.000	—	—
Normal range	1.926	0.877–4.231	0.102
Overweight	3.156	1.251–7.962	0.015
Obesity	3.146	1.212–8.166	0.018

*OR* odds ratio, *95%* *CI* confidence interval

*p*-values were derived from Wald tests

In the sensitivity analysis performed without including BMI as a covariate, the multivariable logistic regression models yielded distinct results for the two outcomes evaluated.

For the low skeletal muscle index (low SMI) outcome, the NRS-2002 showed a borderline association with the outcome after adjustment for age, metastasis, and CNS (OR = 1.788; 95% CI: 1.000–3.195; *p* = 0.050). This finding suggests that the lack of statistical significance observed in the primary model may, at least in part, reflect overadjustment resulting from the inclusion of BMI, a variable that is incorporated into the NRS-2002 score itself. CNS (OR = 0.925; 95% CI 0.487–1.756; *p* = 0.812), age (OR = 1.011; 95% CI 0.994–1.028; *p* = 0.201), and the presence of metastasis (OR = 0.708; 95% CI 0.409–1.225; *p* = 0.217) were not independently associated with the outcome in this alternative model (Table 7).

**Table 7:** Multivariate logistic regression analysis for low SMI excluding BMI

Variable	OR	95% CI	*p*
NRS-2002 (risk)	1.788	1.000–3.195	0.050
CNS (risk)	0.925	0.487–1.756	0.812
Age	1.011	0.994–1.028	0.201
Metastasis	0.708	0.409–1.225	0.217

*p*-values were derived from Wald tests

For the low skeletal muscle radiodensity (low SMD) outcome, neither screening tool demonstrated a significant independent association in the model excluding BMI: NRS-2002 (OR = 1.508; 95% CI 0.800–2.841; *p* = 0.204) and CNS (OR = 1.361; 95% CI 0.676–2.741; *p* = 0.388). Metastasis was also not associated with the outcome (OR = 0.911; 95% CI 0.494–1.679; *p* = 0.765). Age remained the only robust independent predictor (OR = 1.069; 95% CI 1.046–1.093; *p* < 0.001), indicating that each additional year of life increased the odds of low skeletal muscle radiodensity by approximately 6.9%, supporting the well-established phenomenon of age-related myosteatosis (Table 8).

**Table 8:** Multivariate logistic regression analysis for low muscle quality excluding BMI

Variable	OR	95% CI	*p*
NRS-2002 (risk)	1.508	0.800–2.841	0.204
CNS (risk)	1.361	0.676–2.741	0.388
Age	1.069	1.046–1.093	<0.001
Metastasis	0.911	0.494–1.679	0.765

*p*-values were derived from Wald tests

## Discussion

Malnutrition and muscle depletion are frequent and clinically relevant complications in cancer patients, with important implications for treatment tolerance, functional status, and quality of life. In the Brazilian hospital setting, nutritional screening tools are commonly used as the initial, and in many institutions the only, step for identifying patients at nutritional risk, with further comprehensive assessment typically reserved for those classified as at risk. In this real-world cohort of hospitalized oncology patients, CT-based assessment revealed a high prevalence of low muscle mass and impaired muscle quality, frequently occurring even among individuals with preserved or elevated BMI. Taken together, these findings suggest that patients with clinically relevant muscle depletion may not be consistently identified by routine screening procedures, highlighting a potential gap in the current nutritional care pathway and reinforcing the need for more comprehensive assessment strategies [7, 8, 16, 17].^.^

In this study, skeletal muscle depletion was observed in 51.1% of patients assessed by CT, and low skeletal muscle density (SMD) was found in 52.2%. These alterations persisted even among patients with normal or elevated BMI, highlighting the limitations of BMI as a sole marker of nutritional risk. Similarly, Miola et al. reported 73% muscle depletion in head and neck cancer patients evaluated by CT, with over half presenting normal BMI. A systematic review by Prado et al. demonstrated associations between low muscle mass and increased chemotherapy toxicity, reduced survival, and greater side effects [7, 18, 19]. These findings reinforce the need to move beyond BMI as a standalone indicator, favoring more comprehensive approaches that incorporate direct measures of body composition, such as skeletal muscle mass and density.

CT imaging at the L3 vertebra is widely regarded as the reference method for body composition assessment in oncology research. This validated and reproducible method utilizes routinely available staging images without additional patient burden. In this study, CT was crucial for detecting alterations missed by clinical screening: 55% of patients with low muscle mass were not classified as “nutritionally at risk” by screening tools. Ní Bhuachalla et al. similarly found that 41% of sarcopenic patients identified by CT were missed by nutritional screening, and 38% had low muscle mass without significant weight loss, underscoring the difficulty in detecting these conditions via conventional measures. Our findings align with these data, as patients with impaired body composition often showed no changes in anthropometry or subjective criteria [13, 20].

Regarding screening tools, NRS-2002 showed a significant association with SMI, moderate sensitivity, but only 57% overall accuracy. CNS demonstrated higher sensitivity but low specificity, suggesting risk of overestimation and false positives. Previous studies have questioned the NRS-2002’s ability to detect muscle mass alterations specifically, despite its validity for general nutritional risk identification [21–24].

Recent evidence from sarcopenia literature reinforces that screening instruments should be interpreted as case-finding tools rather than diagnostic methods. In a study comparing the original SARC-F with two modified versions (SARC-F-EBM and SARC-F-CalF), Yilmaz et al. demonstrated that although the modified instruments improved diagnostic performance, none achieved sufficient accuracy to replace objective evaluation of skeletal muscle. The authors concluded that screening questionnaires are valuable for identifying individuals who require further assessment, but confirmation should rely on direct measures of muscle mass, muscle strength, and physical performance [25].

Similarly, a recent study evaluating hospitalized older adults showed that combining SARC-F with the Global Leadership Initiative on Malnutrition (GLIM) criteria and calcium and inflammatory biomarkers substantially improved short-term mortality prediction compared with isolated screening approaches. These findings suggest that integrating nutritional screening with objective nutritional, inflammatory, and body composition-related assessments provides a more comprehensive characterization of patient risk than relying on screening tools alone [26]. Although these studies were conducted in older hospitalized populations rather than oncology cohorts, their conclusions are highly consistent with the present findings.

In the present study, the NRS-2002 demonstrated an association with low muscle mass in the bivariate analysis; however, this association did not remain significant after adjustment for BMI in the multivariate analysis. This finding suggests that the previously observed association may be mediated by BMI, a variable that is itself incorporated into the NRS-2002 score. This finding reflects the scope of nutritional screening tools, which identify nutritional risk based on clinical and anthropometric parameters but do not directly incorporate measures of skeletal muscle mass or quality. As a result, clinically relevant muscle depletion, an established component of nutritional risk, may not be captured by these instruments when used in isolation, potentially leading to underestimation of overall risk in this population.

Our findings suggest that patients with higher BMI values, compatible with overweight and obesity, are more likely to present low muscle quality, which is biologically plausible given the greater accumulation of intramuscular fat in these individuals.

Similarly, these results indicate that nutritional screening instruments, regardless of the specific tool applied, are not designed to detect objective alterations in skeletal muscle composition. Given that low muscle mass and poor muscle quality are themselves associated with adverse clinical outcomes and increased nutritional risk, these findings support the need for an additional layer of assessment to complement screening, incorporating objective evaluation of body composition.

Age emerged as an independent predictor of low muscle quality, corroborating the phenomenon of age-related myosteatosis, characterized by the progressive increase in intramuscular fat infiltration. The trend observed for BMI, with higher odds of low muscle quality among overweight and obese individuals, is consistent with the literature and biologically plausible given the greater accumulation of intramuscular fat in these patients.

It is important to note that screening tools like NRS-2002 and CNS assess a combination of factors, including food intake, clinical status, and disease impact; not solely weight loss. This enables early identification of patients at risk for malnutrition or rapid nutritional decline, even before objective body composition changes occur.

Overall, these findings suggest that the absence of an association between nutritional screening tools and body composition abnormalities reflects distinct underlying mechanisms across the two outcomes. For low skeletal muscle index (low SMI), the lack of statistical significance observed in the primary model appears to be partly explained by overadjustment due to the inclusion of BMI. Conversely, for low skeletal muscle radiodensity (low SMD), the absence of an independent association with the screening tools appears to be robust and remains unchanged irrespective of BMI adjustment.

These results are consistent with both international and national literature, indicating that screening tools such as NRS-2002 and CNS are useful for identifying patients who require further nutritional evaluation but may be insufficient to detect all individuals at risk, particularly those with CT-defined alterations in body composition. In this context, CT-based body composition analysis emerges as an important complementary strategy, enabling a more objective and precise assessment of muscle mass and muscle quality, which may support more individualized nutritional and clinical decision-making.

Importantly, in many oncology centers, computed tomography is already routinely performed as part of standard staging, treatment monitoring, and follow-up protocols. Therefore, the incorporation of opportunistic body composition analysis into routine clinical practice may be feasible without requiring additional imaging procedures or increasing patient burden. The use of routinely acquired CT scans to assess skeletal muscle parameters may help clinicians identify patients with occult muscle depletion or myosteatosis who might not be recognized through conventional nutritional screening alone.

From a supportive care perspective, failure to identify patients with altered body composition during hospitalization represents a missed opportunity for early nutritional intervention, symptom management, functional preservation, and optimization of treatment tolerance. Integrating CT-derived body composition assessment into oncology care pathways may contribute to more personalized supportive care strategies, improved risk stratification, and better allocation of multidisciplinary resources in oncology settings.

Although previous international studies have reported discrepancies between conventional nutritional screening tools and CT-derived measurements of muscle mass in oncology populations, data from Brazilian cancer patients remain scarce. Most Brazilian studies involving CT-based body composition analysis have primarily focused on prognostic outcomes, surgical complications, or survival, rather than on the ability of routinely used nutritional screening tools to identify CT-defined alterations in muscle mass and muscle quality.

Therefore, our study adds important real-world evidence from a Brazilian tertiary cancer center by specifically evaluating the relationship between nutritional screening instruments routinely applied in clinical practice and objective CT-derived body composition parameters in hospitalized oncology patients. Considering the epidemiological, nutritional, and socioeconomic particularities of the Brazilian population, these findings contribute to expanding the external validity of the existing literature and reinforce the need for more precise approaches to nutritional assessment in oncology care within low- and middle-income settings.

This study has limitations inherent to its retrospective and cross-sectional design. We acknowledge that selection bias is a relevant concern in studies of this nature. The excluded patients—particularly those admitted to the intensive care unit (ICU) or receiving exclusive palliative care—likely represent a subgroup with more severe nutritional deterioration and poorer functional status. Therefore, the final study population may be biased toward patients with relatively better clinical conditions, potentially underestimating the true prevalence of CT-defined muscle alterations in the broader hospitalized cancer population.

The retrospective design and the constraints of the institutional database did not allow for a systematic comparison between included and excluded patients regarding key clinical variables. This should be considered a limitation of the present analysis, and future prospective studies should aim to implement strategies to minimize selection bias related to exclusion criteria.

Additionally, the single-center design and reliance on available CT scans may limit the generalizability of the findings. Another important limitation is that a formal diagnosis of sarcopenia could not be established, since the retrospective nature of the study precluded the collection of essential variables required by current diagnostic criteria, such as muscle strength and physical performance measures. Therefore, the present analysis focused on CT-defined alterations in muscle mass and muscle quality rather than sarcopenia itself.

Although previous literature has highlighted the importance of integrating muscle strength, functionality, and nutritional assessment in clinical populations, these data were not consistently available in the medical records analyzed. Despite these limitations, the study provides clinically meaningful insights into routine nutritional assessment practices in a real-world oncology setting and contributes novel data from a Brazilian hospitalized cancer population.

## Conclusion

The NRS-2002 demonstrated a significant association with low SMI in the bivariate analysis; however, this association was attenuated and did not reach statistical significance after adjustment for BMI in the multivariable model. Analyses excluding BMI from the model suggest that this attenuation may, at least in part, reflect statistical overadjustment. These findings highlight the importance of complementary body composition assessment strategies, particularly in settings where CT imaging is already available as part of routine oncological care.

Future research should investigate the application of accessible and cost-effective technologies for evaluating muscle mass in hospital settings, thereby facilitating the early detection of muscle depletion.

## Data Availability

The data that support the findings of this study are available from AC Camargo Cancer Center, but restrictions apply to the availability of these data, which were used under license for the current study, and so are not publicly available. Data are, however, available from the authors upon reasonable request and with permission of AC Camargo Cancer Center.
